# Three Signatures of Adaptive Polymorphism Exemplified by Malaria-Associated Genes

**DOI:** 10.1093/molbev/msaa294

**Published:** 2020-11-13

**Authors:** Jacob A Tennessen, Manoj T Duraisingh

**Affiliations:** Harvard T.H. Chan School of Public Health, Boston, MA

**Keywords:** malaria, human population genomics, selection scan, erythrocyte, balancing selection, positive selection

## Abstract

Malaria has been one of the strongest selective pressures on our species. Many of the best-characterized cases of adaptive evolution in humans are in genes tied to malaria resistance. However, the complex evolutionary patterns at these genes are poorly captured by standard scans for nonneutral evolution. Here, we present three new statistical tests for selection based on population genetic patterns that are observed more than once among key malaria resistance loci. We assess these tests using forward-time evolutionary simulations and apply them to global whole-genome sequencing data from humans, and thus we show that they are effective at distinguishing selection from neutrality. Each test captures a distinct evolutionary pattern, here called Divergent Haplotypes, Repeated Shifts, and Arrested Sweeps, associated with a particular period of human prehistory. We clarify the selective signatures at known malaria-relevant genes and identify additional genes showing similar adaptive evolutionary patterns. Among our top outliers, we see a particular enrichment for genes involved in erythropoiesis and for genes previously associated with malaria resistance, consistent with a major role for malaria in shaping these patterns of genetic diversity. Polymorphisms at these genes are likely to impact resistance to malaria infection and contribute to ongoing host–parasite coevolutionary dynamics.

## Introduction

Malaria, a major global infectious disease caused by *Plasmodium* parasites and spread by mosquitoes, has been one of the most important selective pressures on the human lineage (Kwiatkowski 2005). It has been a major cause of mortality in prereproductive humans persistently across several continents for millennia (Carter and Mendis 2002), and thus has greatly impacted human fitness in some populations. Bolstered by the intimate coevolutionary history between humans and *Plasmodium* and the severe pathology of malaria, several of the strongest signatures of selection in the human genome center on genes that impact malaria resistance. These genes include *ABO* (A/B/O blood group; Ségurel et al. 2012, 2013), the cluster of *GYPA*, *GYPB*, and *GYPE* (here abbreviated to *GYPA/B/E*, glycophorin Dantu blood group; Leffler et al. 2017), *ACKR1* (Duffy antigen; Hamblin et al. 2002; King et al. 2011; Chittoria et al. 2012), *CR1* (Knops blood group; Tham et al. 2010; Prajapati et al. 2019), *HBB* (hemoglobin B; Allison 1954; Laval et al. 2019), and *G6PD* (glucose-6-phosphate dehydrogenase; Ruwende et al. 1995; Tishkoff et al. 2001). Malaria remains a major selection pressure to this day, with 200 million cases annually, leading to over 400,000 deaths (Miller et al. 2002; WHO 2019).

The adaptive signatures wrought by *Plasmodium* on humans are useful to characterize and study, for two reasons. First, evolutionary signatures have been critical for finding new malaria-relevant genes (*HBB*, Allison 1954; *GYPA/B/E*, Malaria Genomic Epidemiology Network 2015). There are likely more malaria-relevant genes to be found, as over half of the heritability in malaria resistance (*h*^2^ ∼24%) remains unexplained (Mackinnon et al. 2005; Malaria Genomic Epidemiology Network 2019) and there is substantial geographic heterogeneity in the genetic basis of resistance (Leffler et al. 2017). Progress toward malaria elimination has stalled in recent years, prompting the need for new treatments (White et al. 2014; WHO 2019). Understanding the genetic basis of malaria resistance can pave the way for therapeutics that target host–parasite molecular interactions (Cowman et al. 2017) and inform precision medicine. Second, malaria resistance genes present a robust model system to develop and assess statistical tests for selection, given their striking evolutionary signatures and well-documented phenotypic effects (Malaria Genomic Epidemiology Network 2019). Such tests may be broadly applicable to study other selective pressures, for example, in other host–parasite systems.

Genome-wide scans for selection in humans are now routine (Fan et al. 2016), but they have poor replicability such that different methods produce very different lists of candidate genes. This is true for positive selection (Akey 2009) and may be worse for balancing selection: among six recent studies that scan the genome for balancing selection in Africans or African Americans, the proportion of identified candidate selection targets that are shared between any two scans ranges from 0% to 9% (Andrés et al. 2009; Leffler et al. 2013; DeGiorgio et al. 2014; Siewert and Voight 2017; Bitarello et al. 2018; Cheng and DeGiorgio 2019). This inconsistency may occur because the signature of adaptive variation varies depending on the nature and timing of the selective pressure, and/or it may indicate that many of these signals are spurious. For some genes, selection has been effectively validated phenotypically because allelic effects on infection or fitness have been demonstrated. However, although these known causal genes (e.g., *HBB*, *ABO*, *G6PD*) do show unusual and presumably nonneutral population genetic patterns, they overlap poorly with genome-wide selection scans. Existing tests may be underpowered to detect these true positives, perhaps because selection has acted in subtle ways that do not conform to the assumptions of the tests. There is both a need for analytical tools that can better distinguish biologically meaningful polymorphism from neutral polymorphism, and an opportunity to leverage these functionally validated loci to guide the development of such tools.

**Fig. 1.**
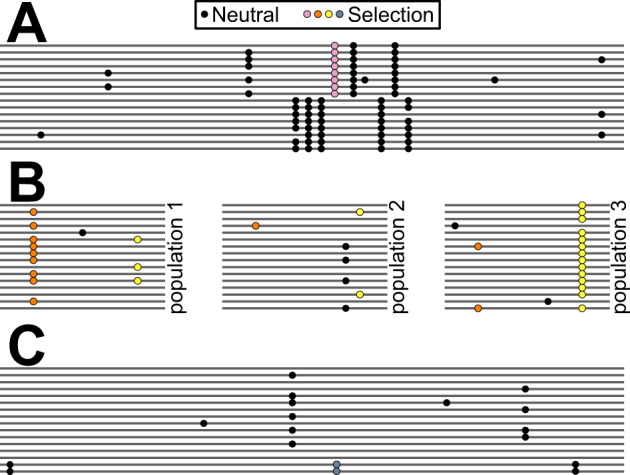
Three complex adaptive scenarios observed more than once among malaria-relevant genes. Lines represent chromosomes. Circles are derived alleles at polymorphic sites (black, neutral; colored, adaptive). (*A*) Divergent Haplotypes. A dense cluster of variants in high linkage disequilibrium occurs within a narrow genomic window surrounding a balanced polymorphism. (*B*) Repeated Shifts. All three pairwise population comparisons show unusually high divergence at one or more variants, suggesting repeated bouts of positive selection. (*C*) Arrested Sweep. A beneficial mutation swept up a long haplotype but stopped at relatively low frequency.

In this paper, we focus on three population genetic patterns that occur more than once in malaria-relevant genes but are poorly approximated by existing statistical tests for selection (table 1 and fig. 1). We call these patterns Divergent Haplotypes, Repeated Shifts, and Arrested Sweeps. Each is represented by two exemplar loci (table 1). From first principles, we can make three predictions about these patterns. First, the patterns should readily distinguish these exemplar loci themselves from the genomic background. Second, the patterns should be enriched among other malaria-relevant genes, and thus, they can help identify novel candidates that may interact with *Plasmodium* in comparable ways. Third, the patterns should also occur among many other instances of adaptive evolution unrelated to malaria, so long as the strength, timing, and spatial heterogeneity of selection have been similar. Here, we develop new ways to summarize population genetic data that readily distinguish these signatures from the neutral background, and assess these statistics using simulations. We then apply our tests to population genomic data (1000 Genomes Project Consortium 2015) to evaluate their ability to detect known malaria-relevant genes and to identify new candidate genes.

**Table 1.** Framework for Detecting Three Adaptive Evolutionary Scenarios.

Scenario	Pattern	Timescale	Exemplar Loci	Best Existing Statistic	New Statistic
Divergent Haplotypes	Variant clusters with high pairwise linkage disequilibrium	10^7^ years ago to present	*ABO*, *GYPA/GYPB/GYPE*	*Z_nS_* (Kelly 1997)	*D_ng_*
Repeated Shifts	Allele frequency shifts in multiple independent populations, yielding high pairwise *F*_ST_	10^5^–10^4^ years ago	*CR1*, *ACKR1*	Parallel *F*_ST_ (Tennessen and Akey 2011)	*T*_R_
Arrested Sweep	Long haplotypes for only the selected allele, low divergence among populations, purifying selection in outgroup	10^4^ years ago to present	*HBB*, *G6PD*	nS_L_ (Ferrer-Admetlla et al. 2014)	*Π*_AHz_

## New Approaches

### Divergent Haplotypes and *D_ng_*

Divergent Haplotypes are observed at *ABO* and *GYPA/B/E*. At these loci, distinct haplotypes have been maintained by balancing selection for millions of years, as evidenced by nonhuman primates sharing the polymorphism (Ségurel et al. 2012; Leffler et al. 2013; Malaria Genomic Epidemiology Network 2015). Within human populations, elevated nucleotide diversity and significantly positive Tajima’s *D* are observed at both *ABO* (Stajich and Hahn 2004) and *GYPA/B/E* (Ko et al. 2011). However, these signals are not strong enough to stand out on a genome-wide scale, and thus, scans for balancing selection fail to identify either of these genes as outliers (Andrés et al. 2009; DeGiorgio et al. 2014; Bitarello et al. 2018), unless they incorporate nonhuman polymorphism (Leffler et al. 2013; Cheng and DeGiorgio 2019). Although transspecies polymorphisms represent strong evidence for selection, they likely constitute a small minority of balanced polymorphisms as they require consistent selection for very long periods across distinct ecological niches. A test based on intraspecies haplotype structure alone may therefore identify additional selection targets.

We developed and evaluated a new test for Divergent Haplotypes (see equations in Materials and Methods). Old, balanced haplotypes accumulate mutations which are protected from genetic drift, leading to increased sequence divergence between the haplotypes (fig. 1*A*). This pattern is often observed in sex-determining regions (Charlesworth 2006), suggesting that any neutral region linked to a balanced polymorphism will have a long coalescence time and show clusters of closely adjacent variants in high linkage disequilibrium (LD). However, older genealogies have also had more time for LD to decay, counteracting the pattern, so any signal can typically be observed only across short genetic distances (DeGiorgio et al. 2014). Because of this potential signal ambiguity of LD, and because calculating LD requires phased data, most tests for long-term balancing selection instead examine the site-frequency spectrum to detect regions where minor allele frequencies (MAF) are usually high and/or similar to each other (Tajima 1989; DeGiorgio et al. 2014; Siewert and Voight 2017). Tests that assess LD directly typically examine long-range LD and are aimed at positive rather than balancing selection (Voight et al. 2006; Sabeti et al. 2007; Ferrer-Admetlla et al. 2014; Garud et al. 2015). The *Z_nS_* statistic examines short-range LD (Kelly 1997), but it is highly sensitive to individual rare variants, which will typically not show high LD with any other variant. Rare variants may or may not be observed depending on stochasticity and sample size, making *Z_nS_* a very noisy statistic. We therefore define a new test statistic which sums across LD correlations rather than averaging them: *D_ng_* (eq. 3). For a target variant, *D_ng_* is the sum of LD correlations with all other variants within a distance of *g* bp. Using *D_ng_*, rare variants have a negligible effect, and the statistic is maximized if there are a large number of variants within a narrow region in high LD with each other. There is no upper limit to *D_ng_*, and its typical range for a given population will depend on overall levels of nucleotide diversity and LD. Therefore, unusual values of *D_ng_* are defined in comparison to the genome-wide average.

### Repeated Shifts and *T*_R_

Repeated Shifts are a form of parallel adaptive divergence, which occurs when positive selection repeatedly causes rapid allele frequency change, resulting in high *F*_ST_ (defined here following Weir and Cockerham 1984), at the same locus in geographically distinct populations (fig. 1*B*). High divergence between independent pairs of populations is unlikely to occur more than once unless driven by natural selection (Tennessen and Akey 2011). Notably, some of the strongest human instances of parallel adaptive divergence occur in genes with large effects on malaria resistance. *ACKR1* is not only the single most divergent gene between Africa and Europe but it is also among the most divergent genes between Europe and Asia. This is due to near-fixation of the Duffy-null allele in sub-Saharan Africa, and independent selection for the Fy^a^ allele in Asia (Hamblin et al. 2002; King et al. 2011; Chittoria et al. 2012). Similarly, *CR1* is also divergent in both Africa–Europe and Asia–Europe comparisons due to positive selection (Prajapati et al. 2019). Our previous genome-wide scan for parallel adaptive divergence (Tennessen and Akey 2011) sought repeated, phylogenetically independent shifts occurring at the same single-nucleotide variant among a set of four populations. Such strict criteria miss *ACKR1* and *CR1*. These genes show changes at different variants in different population comparisons, and these comparisons are nested rather than truly independent (i.e., high Africa v. Eurasia divergence at some variants, and high Africa/Europe v. Asia divergence at other variants). A revised approach might have enhanced power to detect such cases.

We developed and evaluated a new test for Repeated Shifts (see equations in Materials and Methods). Our goal was to detect narrow genomic windows showing unusually high *F*_ST_ in all three pairwise comparisons among three populations, at the same or different variants (fig. 1*B*). Such population triplets are not phylogenetically independent as in Tennessen and Akey (2011), but they cannot be explained without multiple bouts of positive selection. Our approach is based on inversely ranking genomic windows of size *g* bp based on *F*_ST_ and finding windows in which all three pairwise ranks are relatively extreme. Our test statistic *T*_R_ (eq. 4) is a squared average between the lowest (*L*) and highest (*H*) *F*_ST_ ranks scaled by the number of windows examined, which approximates a *P* value reflecting the probability of observing the data if there is no parallel selection acting. However, even values of *T*_R_ that are not individually significant may provide evidence for selection if they are among the most extreme values in the genome.

### Arrested Sweeps and *Π*_AHz_

The Arrested Sweep pattern is observed at *HBB* and *G6PD*. At both of these loci, at least one allele protective against malaria arose in Africa 5–25 ka (Tishkoff et al. 2001; Shriner and Rotimi 2018; Laval et al. 2019) and rapidly increased in frequency. At both loci, the protective allele then stopped spreading and has been maintained at about 10% frequency because it conveys a physiological disadvantage when homozygous/hemizygous (fig. 1*C*). The complete picture is slightly more complex, since these loci harbor more than two alleles with distinct functional effects, which have either arisen independently (e.g., HbC and HbS at *HBB*; Modiano et al. 2001) or occur on the same haplotypic background (e.g., A+ and A− at *G6PD*; Tishkoff et al. 2001; Saunders et al. 2005). However, in most populations, these alternate alleles are rare or in high LD with the main selection target, and thus, the evolutionary scenario can be approximated as a single partial positive selective sweep that has been paused and subsequently maintained by balancing selection.

The resulting population genetic patterns are subtle, and selection has been challenging to detect. The derived allele frequencies are too low to yield intercontinental *F*_ST_ outliers. A sweep leaves a signal of low polymorphism and long-range LD within the beneficial haplotypic lineage (Saunders et al. 2005), but because the sweeps at *HBB* and *G6PD* are several millennia old, LD has begun to decay and to yield a less intense signal at short range. Tests for recent or ongoing partial sweeps seek perfect haplotype homozygosity which is disrupted by such decay, so statistics like iHS (Voight et al. 2006) and XP-EHH (Sabeti et al. 2007) do not readily detect *HBB* or *G6PD*. The related statistic nS_L_ compares mean pairwise haplotype homozygosity length for ancestral and derived alleles. It is significantly nonzero in the 500-kb region surrounding *HBB* (Laval et al. 2019) and a variant within 20 kb of *HBB* is an outlier in a genome-wide nS_L_ scan (Ferrer-Admetlla et al. 2014), suggesting that the partially swept allele is associated with substantially reduced diversity. However, nS_L_ cannot distinguish an arrested sweep from an ongoing positive sweep. Furthermore, nS_L_ and related statistics based on haplotype homozygosity are also sensitive to relatedness among samples and new mutations or sequencing errors that disrupt the otherwise perfect similarity among haplotypes. Tests for soft sweeps expect more than one haplotype to have rapidly increased in frequency, a slightly different scenario (Garud et al. 2015). Similarly, because of the low frequency and recent origin of the derived allele, these genes lack many classic signatures of balancing selection like intermediate frequency alleles (e.g., Tajima’s *D*; Tajima 1989) or ancient haplotypes (e.g., *Z_nS_* or transspecies polymorphisms). Because the expected frequency of minor allele homozygotes would be low even if neutral, there is often insufficient power to detect a deviation from Hardy–Weinberg equilibrium. Thus, despite being canonical examples of adaptive polymorphism, these genes are almost never detected in genome-wide scans for partial sweeps or balancing selection (Voight et al. 2006; Akey 2009; Andrés et al. 2009; Leffler et al. 2013; DeGiorgio et al. 2014; Siewert and Voight 2017; Bitarello et al. 2018; Cheng and DeGiorgio 2019).

We developed and evaluated a new test for Arrested Sweeps (see equations in Materials and Methods). This test seeks recently arisen variants which are beneficial when heterozygous but strongly deleterious otherwise, as with *HBB* and *G6PD* (fig. 1*C*). This test is conducted on a single target population. It also requires several other populations hypothesized to experience similar selective pressures; along with the target population, these constitute the “ingroup.” Finally, it requires an “outgroup” population in which there is no heterozygote advantage to the derived variant, only the deleterious effect (as with any population where malaria does not occur). There are two evolutionary components of an Arrested Sweep: the sweep (positive selection) and the arrest (balancing and purifying selection).

The first evolutionary step is a partial positive selective sweep. Even if LD has started to decay, the signal of a sweep may extend beyond the range of haplotype homozygosity, in the form of reduced, but not necessarily nonzero, nucleotide diversity linked to the swept haplotype. In other words, individuals homozygous for a swept allele have fewer total heterozygous sites than individuals homozygous for the ancestral allele. The second evolutionary step, which distinguishes an Arrested Sweep from an ongoing partial sweep, is balancing selection maintaining the polymorphism at the optimal frequency in the ingroup while purifying selection excludes it from the outgroup. Assessing this signal starts with *F*_ST_ within the ingroup. An arrested sweep maintained at constant frequency by balancing selection will show low *F*_ST_ among populations that are experiencing the same selection pressure. An ongoing sweep should not show this pattern and may even show unusually high *F*_ST_ if the sweep has proceeded farther in some populations than others. In addition, purifying selection acts in the outgroup where there is no heterozygote advantage, so the MAF in the outgroup should be very close to zero. Our final test statistic *Π*_AHz_ (eq. 7) is a product of metrics that capture these steps: positive sweep in the target population, balancing selection across the ingroup, and purifying selection in the outgroup. There is no upper limit to *Π*_AHz_ and unusual values of *Π*_AHz_ are defined in comparison to the genome-wide average.

## Results

### *D_ng_* in Simulations

Simulation results show that *D_ng_* values are typically higher for balanced polymorphisms, relative to neutral polymorphisms, under a wide range of parameters (supplementary fig. 1, Supplementary Material online). Intermediate *g* values (500–1,000 bp) were optimal to minimize the overlap between selection and neutral windows. The power of *D_ng_* is maximized when recombination rates are low, the polymorphism is old, and mutation rates surrounding balanced polymorphisms are similar to those in neutral regions. Skewing the expected MAF had little effect, in contrast to many common tests for balancing selection that seek intermediate-frequency variants. For *g* of 500 and human-relevant parameters, variants with approximately *D_ng_* ≥ 10 should be enriched for true balanced polymorphisms, though with inevitable false positives and false negatives.

### *D_ng_* in Human Population Data

**Fig. 2.**
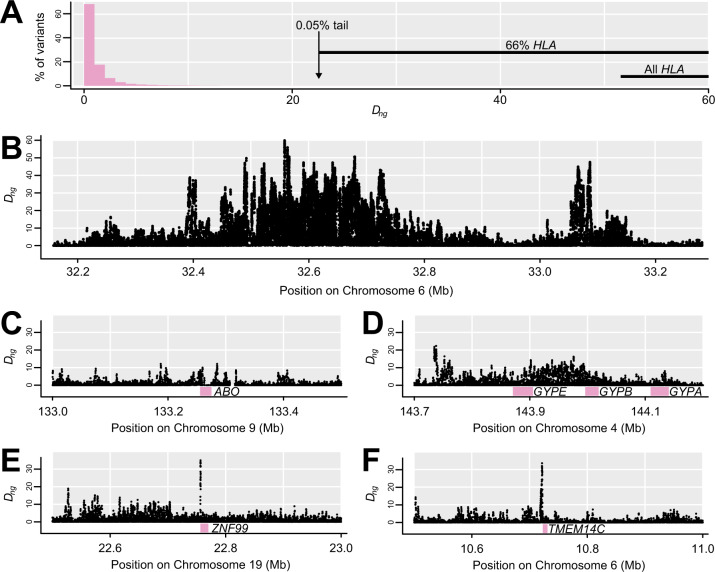
Distribution of *D_ng_*, including notable genomic outliers. (*A*) Histogram of *D_ng_* shows that the vast majority of variants have low values, and the 0.05% which exceed 22.6 are enriched for variants in or near the HLA. (*B*) This section of the HLA region shows the highest *D_ng_* values in the genome. (*C*) *ABO* slightly surpasses *D_ng_* of 10 within the coding region. (*D*) Noncoding regions near *GYPA/B/E* show high *D_ng_*. (*E*) The highest *D_ng_* in or near a gene occurs just upstream of *ZNF99* (table 2). (*F*) One of the highest *D_ng_* sites in or near a gene occurs just upstream of *TMEM14C* (table 2).

In our focal population YRI (Yoruba in Ibadan, Nigeria), *D_ng_* with *g* of 500 ranged from 0 to 59.8 (median = 0.2, 95% interval = 0.0–5.9; fig. 2*A*). Only 0.56% of variants had *D_ng_* ≥ 10. Variants in or near the HLA accounted for 15% of variants with *D_ng_* ≥ 10, a majority (66%) of variants in the top 0.05% (*D_ng_* ≥ 22.6) and the 58 highest variants (fig. 2*B*). There are 243 coding genes with at least one exonic *D_ng_* value ≥10, including *ABO* (fig. 2*C*). The *GYPA/B/E* cluster does not show a strong exonic signal but many high-*D_ng_* variants, including two reaching the top 0.05% threshold, occur in the intergenic region between *GYPE* and *FREM3* where the strongest signal of selection and disease-association of *GYPA/B/E* has previously been detected (Leffler et al. 2013; Malaria Genomic Epidemiology Network 2015; fig. 2*D*). These results suggest that *D_ng_* does capture the intended empirical selection signal. The top 50 genes outside of the HLA region (table 2 and supplementary table 1, Supplementary Material online), based on *D_ng_* in exons or within 1 kb upstream, all showed *D_ng_* >15.5 (top 0.15% of variants). The most extreme genes include *ZNF99* (fig. 2*E*) and *TMEM14C* (fig. 2*F*).

**Table 2.** Top Five Genes for Each Statistic When Occurring in Exons or Within 1 kb Upstream, Excluding the HLA Region.

Gene	Chromosome	Statistic	Value	Description
*ZNF99*	19	*D_ng_*	34.97	Zinc finger protein, possible role in viral infection
*SNX29*	16	*D_ng_*	34.92	Sorting nexin, may regulate intracellular trafficking
*CYP2B6* ^a^	19	*D_ng_*	32.56	Cytochrome P450 family enzyme, role in metabolizing xenobiotics
*TMEM14C* ^a^	6	*D_ng_*	28.77	Heme precursor transporter, role in erythropoiesis
*KRTAP9-8*	17	*D_ng_*	28.47	Keratin associated protein with role in hair structure
*PTK6*	20	*T*_R_	1.54e-08	Cytoplasmic nonreceptor protein kinase, epithelial signaling role
*SRMS*	20	*T*_R_	3.42e-07	Tandem paralog of *PTK6*
*FBXO31*	16	*T*_R_	8.85e-07	F-box protein with regulatory role
*SPNS2* ^a^ ^,b^	17	*T*_R_	9.10e-07	Transporter of sphingosine 1-phosphate
*TTLL10*	1^c^	*T*_R_	1.03e-06	Polyglycylase
*RLIM*	X	*Π*_AHz_	495.12	Zinc finger protein transcription regulator
*ABCB7* ^a^	X^c^	*Π*_AHz_	272.91	Heme transporter, role in erythropoiesis
*HBB* ^a^ ^,b^	11	*Π*_AHz_	194.83	Oxygen-transport metalloprotein in erythrocytes
*UGT2B10*	4	*Π*_AHz_	187.49	Liver glycosyltransferase, role in metabolizing xenobiotics
*RABGAP1L* ^a^	1^c^	*Π*_AHz_	162.56	GTPase-activating protein, regulatory role in hematopoiesis

a Associated with erythrocytes or erythroid cells (text mining *Z* score ≥2 and/or in proteome).

b Correlated with malaria in genome-wide association studies (GWAS).

c Low-recombination region.

### *T*_R_ in Simulations

Among 10,000 neutral simulated windows, the distribution of *T*_R_ approximates a uniform distribution of expected *P* values (supplementary fig. 2, Supplementary Material online). The fit is poor for higher values of *T*_R_, but since the practical question is whether low *T*_R_ values are lower than would be expected by chance, this is unimportant. Upon adding a single window showing parallel adaptation to this set of 10,000, the adaptive window typically shows the lowest *T*_R_. With a selection coefficient of 0.05, most (65%) adaptive windows have lower *T*_R_ than all neutral windows, and 45% are individually significant (*T*_R_ < 0.05/10,001), even though only 4% show *F*_ST_ higher than all neutral *F*_ST_ values for all three pairwise comparisons. Therefore, combining *F*_ST_ values into *T*_R_ provides higher power to detect adaptation than individual pairwise *F*_ST_. With a selection coefficient of 0.005, both *T*_R_ and *F*_ST_ are similar to their neutral distributions and only 8% of adaptive windows show *T*_R_ < 0.05/10,001, demonstrating the limits of *F*_ST_-based tests when selection is weak. Increasing the mutation rate or including background selection has a negligible effect on the neutral distribution (supplementary fig. 2, Supplementary Material online).

### *T*_R_ in Human Population Data

**Fig. 3.**
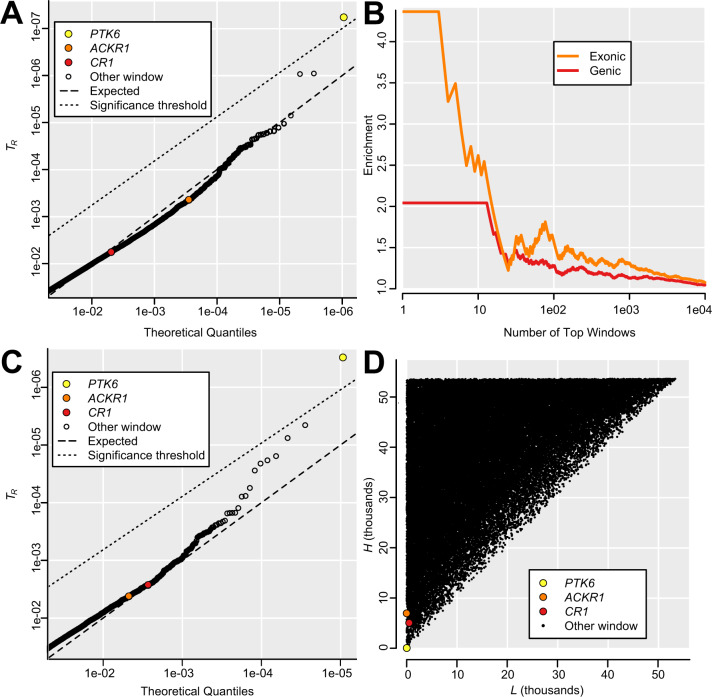
Genomic outliers for *T*_R_. (*A*) Partial QQ plot showing *T*_R_ less than 0.05, representing 7% of genomic windows, for *g *=* *5 kb. A single window overlapping *PTK6* surpasses the significance threshold, whereas most other windows are close to the expected neutral distribution. Although windows overlapping *ACKR1* and *CR1* are not individually significant, they occur among the 0.5% most extreme windows. (*B*) 5-kb windows with low *T*_R_, shown for continuously increasing thresholds, are enriched for windows that overlap genes, and even more so for windows that overlap exons, consistent with *T*_R_ capturing phenotypically relevant polymorphism. This effect cannot be explained by background selection in genes, which would slightly increase *T*_R_ (supplementary fig. 2*D*, Supplementary Material online). (*C*) Partial QQ plot showing *T*_R_ less than 0.05, representing 8% of genomic windows, for *g *=* *50 kb. As in (A), a window overlapping *PTK6* is individually significant, whereas *ACKR1* and *CR1* are among the top outliers. (*D*) Lowest rank *L* (most extreme *F*_ST_) and highest rank *H* (least extreme *F*_ST_) for *g *=* *50 kb, highlighting genes in outlier windows.

In populations from Africa, Europe, and Asia, *T*_R_ with *g* of 5 kb could be estimated for over 520,000 windows, representing over 2.6 Gb. The distribution of *T*_R_ closely approximated a uniform neutral distribution of *P* values, with 7% of windows showing *T*_R_ less than 0.05. The median number of common variants (MAF ≥5%) in these low-*T*_R_ windows was 16, similar to the genome-wide median of 14. Only a single window, overlapping the majority of the coding sequence of gene *PTK6*, had an individually significant *T*_R_ (less than corrected α of 1e-07; fig. 3*A*). However, windows with low *T*_R_ are good candidates for Repeated Shifts, even if not individually significant. Windows overlapping *ACKR1* occurred in the 0.03% most extreme windows (*T*_R_ = 0.0004), whereas windows overlapping *CR1* occurred in the 0.5% most extreme windows (*T*_R_ = 0.0057). Low-*T*_R_ outliers are enriched for genic and exonic windows (fig. 3*B*), consistent with *T*_R_ capturing adaptive variation. After staggering window starting positions to capture all outliers, the top 50 genes have an exon overlapping at least one window with *T*_R_ under 0.0002 (supplementary table 2, Supplementary Material online). Outliers are clustered in the genome such that all windows with *T*_R_ under 0.0002 can be grouped into 52 meta-windows under 1 Mb in size (median = 8.5 kb; range = 5–946 kb; cumulative = 3.997 Mb; minimum distance between meta-windows = 1.8 Mb), and thus, several outlier genes probably reflect the signal at linked genes (e.g., *SRMS* and *PTK6*, table 2) Results were largely similar with *g* of 50 kb (fig. 3*C* and *D*), indicating that *T*_R_ is robust to the choice of window size. Because these wider windows often overlap more than one gene, complicating interpretation, we focus on results with *g* of 5 kb (table 2 and supplementary table 2, Supplementary Material online).

### *Π*_AHz_ in Simulations

In simulations, *Π*_AHz_ is substantially higher under an Arrested Sweep than under neutrality (supplementary fig. 3, Supplementary Material online). Under strong selection (heterozygote fitness = 1.09), most simulations produced higher *Π*_AHz_ (median = 43; 95% interval = 17–78) than neutral simulations (median = 1; 95% interval = 0.01–32). *Π*_AHz_ is calculated from several unrelated metrics, and each component has a distribution under selection that differs from the neutral distribution, resulting in a statistic that is very sensitive to Arrested Sweeps. *Π*_AHz_ was also elevated under weak selection (heterozygote fitness = 1.01), but the effect was relatively modest (median = 10; 95% interval = 0.2–45). *Π*_AHz_ was slightly elevated under reduced recombination rate (median = 6; 95% interval = 0.07–165) or increased mutation rate (median = 3; 95% interval = 0.04–64). In practice, these results suggest that under similar parameters, variants with *Π*_AHz_ ≥ 20 should be enriched for true balanced polymorphisms, whereas variants with *Π*_AHz_ ≥ 100 are very unlikely to be neutral, with the caveat that nonneutral processes can in rare instances also produce similarly high *Π*_AHz_.

### *Π*_AHz_ in Human Population Data

**Fig. 4.**
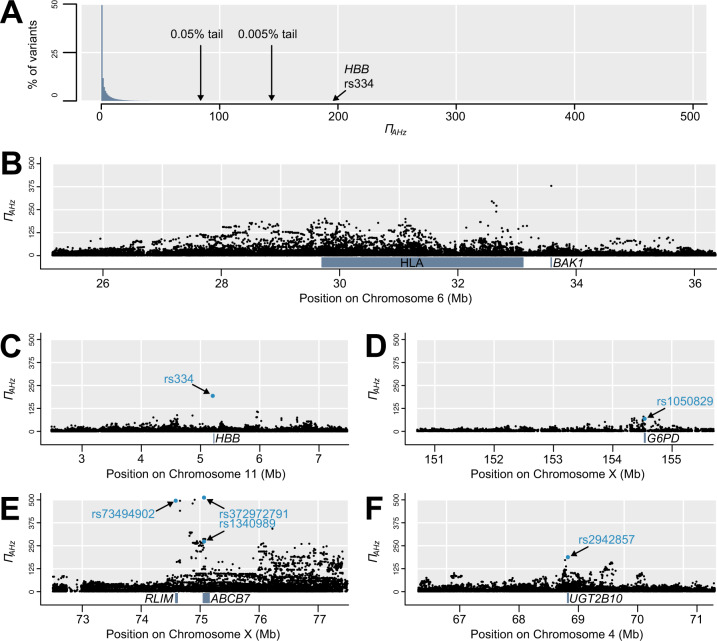
Distribution of *Π*_AHz_, including notable genomic outliers. (*A*) Histogram of *Π*_AHz_ shows that the vast majority of variants have low values, though the sickle-cell polymorphism rs334 occurs in the 0.005% tail. (*B*) High *Π*_AHz_ occurs throughout the HLA region and this signal extends beyond its borders, including in *BAK1*. (*C*) Sickle-cell polymorphism rs334 in *HBB* shows exceptionally high *Π*_AHz_. (*D*) Several variants in or near *G6PD* show high *Π*_AHz_, including disease-linked missense variant rs1050829. (*E*) The highest *Π*_AHz_ values in the genome by far occur in this section of the X chromosome, with the highest variants in *RLIM* and *ABCB7*. (*F*) Splice-acceptor variant rs2942857 in *UGT2B10* is the highest exonic autosomal variant after rs334.

Within our focal population YRI, using all five sub-Saharan African populations to calculate *F*_ST_ and MAF, and Europe as the outgroup, *Π*_AHz_ ranged from 0 to 511.2 (median = 1.0, 95% interval = 0.0–23.8; fig. 4*A*). The HLA region accounted for 4% of autosomal variants in the top 0.05%, with additional outliers closely linked to it including the top autosomal variant in an intron of *BAK1* (fig. 4*B*), but this HLA enrichment was much less pronounced for *Π*_AHz_ than it was for *D_ng_*. Our target genes *HBB* and *G6PD*, and specifically their phenotype-associated nonsynonymous polymorphisms, showed very high *Π*_AHz_ and are among the most extreme outliers. In *HBB*, the Glu-Val missense variant rs334 that causes sickle-cell anemia shows *Π*_AHz_ of 194.8, placing it in the top 0.005% of all variants (fig. 4*C*). Only 144 variants in the entire genome have a higher *Π*_AHz_ than rs334. If variants on chromosomes 6 (HLA) and X (see below) are ignored, rs334 remains among the top 14 variants, and the only one within a protein-coding gene. In *G6PD*, the Asn-Asp missense variant rs1050829 (allele A+), associated with G6PD deficiency and in high LD with malaria resistance variant rs1050828 (allele A−) and several other variants, shows *Π*_AHz_ of 68.8, placing it in the top 0.5% of all variants, both on the X chromosome and genome-wide (fig. 4*D*). The top 50 genes outside of the HLA region (supplementary table 3, Supplementary Material online and fig. 4*C*, *E*, and *F*), based on *Π*_AHz_ in exons or within 1 kb upstream, all exceed *Π*_AHz_ of 99.5 (top 0.025% of variants). This list includes several genes on chromosome 6 that could reflect the effect of HLA selection, as its signal appears to extend for several megabases surrounding the HLA (fig. 4*B*). Although *HBB* is the highest autosomal gene, it is exceeded more than 2.5-fold by two adjacent X-linked genes, *ABCB7* and *RLIM* (fig. 4*E*). The *ABCB7* signal includes Ala-Val missense variant rs1340989 (*Π*_AHz_ = 272.9) and intron variant rs372972791 with the highest observed *Π*_AHz_ of 511.2. *ABCB7* is the peak of a 3-Mb region from X positions 74.5–77.5 Mb, with more than 100 variants showing *Π*_AHz_ over 200, a threshold that excludes all other X-linked variants and all but 17 autosomal variants. After rs334, the second highest exonic autosomal outlier is a splice-acceptor variant in *UGT2B10* (fig. 4*F*).

### Synthesis

**Fig. 5.**
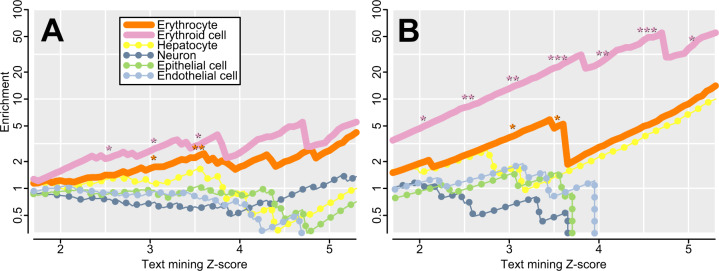
Enrichment for genes associated with various cell types, with higher *Z* scores indicating a stronger association via text mining (Santos et al. 2015). At intervals of 0.5, significant enrichments are indicated (*P < 0.05; **P < 0.01; ***P < 0.001). Genes associated with erythrocytes and erythroid cells are significantly enriched (solid lines), and enrichment increases with score, suggesting a prevalence of genes with particularly high specificity to red blood cells. Enrichment is higher among erythroid cell genes than among erythrocyte genes, suggesting that many outliers are more important in erythroid progenitors than in mature cells. No significant enrichment is observed in control tissues (dotted lines). (*A*) The top 150 genes for the three tests (supplementary tables 1–3, Supplementary Material online). (*B*) The top fifteen genes for the three tests (table 2).

Among the top 50 outliers for each test, there are no GO terms with significant FDR values. We specifically tested for genes important to red blood cells, given their centrality to our exemplar genes and to *Plasmodium* invasion, and we observe substantial enrichment. Genes with a proteomic presence in erythrocytes are abundant, but not significantly so (1.3-fold enrichment; 23 genes; *P* = 0.10; Bryk and Wiśniewski 2017). There is significant enrichment for erythroid and erythrocyte genes as detected by text mining for associations between genes and words in Medline abstracts (Santos et al. 2015), both among the top 50 outliers for each test (e.g., 1.7-fold enrichment with 19 of 150 genes at *Z* ≥ 3; *P* = 0.015; supplementary tables 1–3, Supplementary Material online and fig. 5*A*), and among the top five outliers for each test (e.g., 4.5-fold enrichment with 5 of 15 genes at *Z* ≥ 3; *P* = 0.0035; table 2 andfig. 5*B*). Enrichment is even greater among genes with higher text mining scores, for which there is stronger evidence for importance in red blood cells (fig. 5). Independent of this analysis, our top hits are significantly enriched for correlations with malaria susceptibility in genome-wide association studies (“GWAS”; 2.7-fold enrichment; 9 of 150 genes; *P* = 0.007; supplementary tables 1–3, Supplementary Material online). These nine GWAS hits include known exemplar gene *HBB* and adjacent gene pair *METTL7B* and *ITGA7* that share a signal with each other, but even if *HBB* is discarded and the adjacent pair is merged, the enrichment is still significant for seven matches (*P* = 0.048). We do not see an enrichment for proteins previously shown to interact with *Plasmodium* or piroplasmid parasites (*P* = 0.88; Ebel et al. 2017). Many genes are observed both among our outliers and among those of nine previous genome-wide scans for selection using various tests (supplementary table 4, Supplementary Material online). There is a trend toward enrichment with all nine previous scans, though it is not always significant. Notable comparisons include five genes identified by both *D_ng_* and a composite likelihood scan for balancing selection (DeGiorgio et al. 2014; 13.4-fold enrichment, *P* = 3.5e-05), thirteen genes identified by both *T*_R_ and the parallel adaptive divergence scan at the level of individual variants (Tennessen and Akey 2011; 4.1-fold enrichment; *P* = 9.4e-06), and two genes identified by both *Π*_AHz_ and iHS (Voight et al. 2006; 2.9-fold enrichment; *P* = 0.15). Finally, across all tests, we see an enrichment for low-recombination regions of the genome (2.5-fold enrichment; 20 of 150 genes; *P* = 0.0001), especially for *Π*_AHz_ which encompasses long-range LD (table 2 and supplementary tables 1–3, Supplementary Material online). Excluding the low-recombination genes has little effect on our conclusions (e.g., 1.9-fold enrichment for erythroid and erythrocyte genes with 18 of 130 genes at *Z* ≥ 3; *P* = 0.007), and because our *Π*_AHz_ exemplar gene *G6PD* occurs in a low-recombination region it is plausible that outliers in these regions represent true positives.

## Discussion

### Three New Statistical Assays for Nonneutral Polymorphism

We present three novel summary statistics that reflect evidence for selective nonneutrality in population genetic data. There are many such statistical tests (Vitti et al. 2013), which are frequently employed to find putative targets of selection across the human genome (Sabeti et al. 2007; Akey 2009; Fan et al. 2016). However, despite this plethora of statistical tools, it remains challenging to conclusively identify instances of positive or balancing selection in humans. Genes which are known to behave nonneutrally, either because fitness or phenotypic impact of genotypes have been measured directly (e.g., overdominance at *HBB*; Aidoo et al. 2002), or because of evidence from other species (e.g., transspecies polymorphisms at *ABO* and *GYPA/B/E;*Ségurel et al. 2012; Leffler et al. 2013), are not necessarily outliers in scans for selection across human populations. Here, we focused on genes with polymorphisms already known to be adaptive and shaped by infectious disease, and we attempted to capture unusual empirical patterns at these genes that are plausibly driven by selection in accordance with population genetic theory. All of our tests require more than one atypical pattern to occur simultaneously, which should reduce false positives: extreme *D_ng_* requires an unusual density of variants and also high LD among them, extreme *T*_R_ requires two separate instances of unusually pronounced *F*_ST_, and *Π*_AHz_ requires separate signals of positive, balancing, and purifying selection. However, as with other tests of nonneutrality, the actual false positive rate is difficult to assess, and the best candidate outliers are those with independent support such as detection by multiple tests (Akey 2009; supplementary table 4, Supplementary Material online) or functional validation. Our statistics are inspired by patterns at malaria-relevant genes in humans, and some of the novel outliers may also be relevant to malaria. However, malaria is not the only selective pressure capable of producing these patterns, and these tests are potentially applicable to detecting selection due to other causes and/or in other species.

Both simulated and empirical results suggest that these tests are robust. However, they carry several caveats and limitations. Like other outlier-based tests (Akey 2009), these tests are intended to identify genomic regions that are most suggestive of certain hypothesized modes of selection, under the a priori assumption that a small proportion of the genome did evolve according these modes. They are not intended to test a null hypothesis of complete neutrality, and there are no clearly defined thresholds beyond which neutrality can be rejected. None of the tests employ outgroup species and so are naïve with respect to derived or ancestral status of alleles, but if selective pressures fluctuate and act on standing variation then even an ancestral allele could be adaptive. As with most selection scans, these tests assume that mutation and recombination rates across the genome are similar; as this assumption is typically violated, their power may vary across genomic regions. Each test also carries its own specific caveats. For Divergent Haplotypes, selectively neutral processes like gene conversion or introgression (e.g., from archaic hominids; Ragsdale and Gravel 2019) could lead to high sequence divergence between haplotypes and thus elevated *D_ng_*, as could misaligned reads from paralogs. Furthermore, the most polymorphic genomic regions can be poorly represented in population genomic data sets if high sequence divergence and large indels impede genotyping, so some of the strongest Divergent Haplotypes signatures could be missed by *D_ng_* in practice. For Repeated Shifts, *T*_R_ approximates a *P* value but is not formally a *P* value for a defined null hypothesis. *F*_ST_ depends on the MAF, and single-variant-based tests for parallel *F*_ST_ require considerable filtering based on allele frequency (Tennessen and Akey 2011). However, since *T*_R_ considers *F*_ST_ across numerous variants within a window, typically showing a wide range of MAFs, it should be largely robust to this effect. *T*_R_ is unlikely to be influenced by differences in background selection (Matthey-Doret and Whitlock 2019; supplementary fig. 2*D*, Supplementary Material online). For Arrested Sweeps, *Π*_AHz_ carries the assumption that differences in heterozygosity are caused by a shared physical and evolutionary association with the target locus. This assumption is violated if there is LD not caused by physical linkage (i.e., due to family or population structure), or if LD due to sampling error is substantial enough to affect the true LD signal. The latter can occur when regions of extraordinarily high polymorphism or haplotype structure such as a centromere or the HLA (fig. 4*B*) are included in the window used to estimate heterozygosity. *Π*_AHz_ depends on selection acting consistently one way in the ingroup and another way in the outgroup, which may not always be the case. All of these statistics can best be thought of as tools for identifying candidate genes, but follow-up study is required before drawing firm conclusions about evolutionary history or functional impact.

The tests detect the six exemplar loci which motivated this study, but with varying degrees of success. Most strikingly, *Π*_AHz_ is a near-perfect test for an *HBB*-like signal, as *HBB* is the third most extreme gene and the top autosomal gene. *ACKR1* and *G6PD* are also notable outliers, falling in the top 1% of genes for their respective statistics. The remaining three exemplar loci are less extreme outliers, but all fall within the top 5% of genes for their respective statistics. As noted above, *D_ng_* may be underpowered if some polymorphisms are absent from the data set, as is the case for *ABO* and* GYPA/B/E*, which could partially explain why this statistic was the least powerful at detecting its exemplar loci. Other loci are also strongly associated with malaria but would have made poor exemplar genes in this analysis and were therefore ignored. For example, *Plasmodium* has driven an Arrested Sweep on *SLC4A1* in Southeast Asia (Paquette et al. 2015), but these populations are poorly represented in the 1000 Genomes. In addition, though *ATP2B4* is globally associated with malaria (Malaria Genomic Epidemiology Network 2019), evidence for nonneutral evolution is mild and/or very geographically localized (Gelabert et al. 2017; Gouveia et al. 2019). Indeed, *ATP2B4* was not a notable outlier in any test (*D_ng_* = 5.98, *T*_R_ = 0.0094, *Π*_AHz_ = 6.94).

### Adaptation to Malaria

Malaria is caused by *Plasmodium*, which for millions of years has been a parasite of hominid primates and other vertebrates. Malaria has been one selection pressure on our six exemplar genes, but others may have had equal or greater importance, such as gut pathogens on *ABO* (Ségurel et al. 2012) and tuberculosis on *CR1* (Noumsi et al. 2011). Genetic variants which conveyed resistance to ancient *Plasmodium* parasites in our prehuman ancestors, if not fixed long ago, persist as balanced polymorphisms with a Divergent Haplotypes signature at loci like *ABO* and *GYPA/B/E*. The Repeated Shifts pattern is tied to the emergence of modern *Plasmodium* species from parasites of nonhuman apes, including *P. vivax* probably over 40,000 years ago and *P. falciparum* within the past 10,000 years (Loy et al. 2017; McManus et al. 2017; Daron et al. 2020), which have shaped variation at *ACKR1* (Hamblin and Di Rienzo 2000; King et al. 2011; Chittoria et al. 2012; Loy et al. 2017) and *CR1* (Tham et al. 2010; Prajapati et al. 2019). Arrested Sweeps are even more recent and are associated with the expansion of agriculturally facilitated mosquito habitat across sub-Saharan Africa during the past few thousand years, causing the spread of *P. falciparum* and consequently of resistance alleles at *HBB* and *G6PD* (Tishkoff et al. 2001; Shriner and Rotimi 2018; Laval et al. 2019).

We observed an enrichment for malaria-associated chromosomal regions from three large GWAS (Timmann et al. 2012; Malaria Genomic Epidemiology Network 2019; Milet et al. 2019), especially among *T*_R_ outliers (supplementary table 2, Supplementary Material online). Two particularly promising candidates are *PTPRM* and *MYLK4*, which are among the very strongest candidates for recurrence of mild malaria attacks in infants (Milet et al. 2019). In particular, *PTPRM* was the top prioritized gene in the GWAS based on functional consequences (*P* = 3.8e-08; Milet et al. 2019), and the top disease-associated variant in the gene occurs in between the two variants that define the Repeated Shifts signal (*T*_R_ = 2e-04), just upstream of an alternate transcript start. Furthermore, less than 2 kb upstream of malaria-associated *SPNS2*, the fourth-highest *T*_R_ outlier (table 2), there are three variants within 36 bp of each other that have undergone three distinct allele frequency shifts in Africa, Asia, and Europe. Finally, adjacent genes *METTL7B* and *ITGA7* share a Repeated Shifts signal and a GWAS signal and are both overexpressed during severe malaria (Lee et al. 2018). Any gene at the intersect of selection signal and phenotype association is worthy of further consideration, as such combined evidence has been instrumental in implicating known loci like *GYPA/B/E* (Malaria Genomic Epidemiology Network 2015).

### Erythrocytes and Erythropoiesis

We observe a pronounced enrichment for genes with a role in red blood cells (fig. 5). Our exemplar genes all act during this stage, and new outliers may be under similar selection pressures. There are two principal mechanisms by which such genes may impact blood-borne parasites. In the first mechanism, erythrocyte surface proteins encoded by transmembrane genes like *ACKR1*, *CR1*, *ABO*, *GYPA*, and *GYPB* act as receptors during parasite invasion (Cowman et al. 2017) or mediate cytoadherence (Cserti and Dzik 2007). Several such receptors remain to be discovered (Cowman et al. 2017), but there are few compelling candidates among our novel outliers. The strongest contender is *RHD* (*T*_R_ = 6e-05; fig. 3), which encodes the transmembrane D antigen for the Rh blood group, a component of the erythrocyte cell surface connected to *Plasmodium* invasion (Chung et al. 2007). Remarkably, *RHD* also shows a moderate Divergent Haplotypes signature within its coding region (*D_ng_* = 13.2, higher than either of the Divergent Haplotypes exemplar genes), suggesting it could have been subject to both adaptive processes. However, unlike at *ACKR1* and *CR1*, the *RHD* signal involves Europe-specific divergence and thus shows a slightly different evolutionary history, perhaps driven by other parasites (Novotná et al. 2008). Other outliers also encode transmembrane proteins, but there is little evidence that they are expressed on the surface of mature erythrocytes.

The second principal mechanism of resistance is to alter erythrocyte development and cellular integrity. Such changes can affect intracellular parasite growth and survival, though perhaps with reduced function or a similar cost to the host. Hemoglobinopathies and enzymopathies that protect against malaria are conveyed by *HBB*, *G6PD*, and other loci like *FECH* (Taylor et al. 2013; Smith et al. 2015). Across all three tests, several of our top outlier loci play keys roles in erythropoiesis (table 2 and supplementary tables 1–3, Supplementary Material online), and thus, we observe more evidence for adaptation via this mechanism than via the first mechanism of invasion receptors. Loci involved in erythroid development are not necessarily expressed in the mature proteome (Bryk and Wiśniewski 2017), and thus, we see greater enrichment for (precursor) erythroid cell genes than for (mature) erythrocyte genes (fig. 5). In addition to *HBB*, our most extreme outliers include *TMEM14C* and *ABCB7*, both implicated in erythroid maturation (Conte et al. 2015; table 2). *TMEM14C* encodes a transmembrane protein essential for erythroid synthesis of heme (Yien et al. 2014), and many of the highest *D_ng_* variants occur in a 4.5-kb region in its upstream cis-regulatory region (*D_ng_* = 28.8, fig. 2*F*). Beyond the *D_ng_* signal, variants near *TMEM14C* are also among the most differentiated between Europeans and Africans genome-wide (*F*_ST_ > 0.8) and may underlie the pronounced differences in *TMEM14C* expression between these continents (Quach et al. 2016). *ABCB7*, a transmembrane iron transporter in the heme pathway, is essential for erythropoiesis and causes anemia when deficient (*Π*_AHz_ = 272.9; Pondarre et al. 2007; Severance and Hamza 2009; fig. 4*E*). The outlier region centered on *ABCB7/RLIM* far exceeds *Π*_AHz_ in the rest of the genome. Recombination is unusually low in this region and this can increase *Π*_AHz_ (supplementary fig. 3, Supplementary Material online), which may explain the magnitude of the signal. Like *TMEM14C* and *ABCB7*, several outliers are directly involved in heme binding and/or biosynthesis (*PRDX1*, *UROD*, *CYB5R3*; Fermo et al. 2008; Severance and Hamza 2009), whereas liver-expressed *UGT2B10* is part of a glycosyltransferase family that catalyzes heme breakdown (Sticova and Jirsa 2013; fig. 4*F*). Other outliers have roles in hematopoiesis (*RABGAP1L*, Roberti et al. 2009; *MAP1LC3B*, Kang et al. 2012) or erythrocyte morphology (*MYH9*, Smith et al. 2019). Although the potential link to malaria is intriguing, other infectious agents also interact with red blood cells and may have had a comparable impact on these selection signatures.

### Adaptation to Infectious Disease beyond Malaria and the Red Cell

Beyond blood parasites, our outliers likely reflect various important selective pressure in humans, possibly including climate, diet, environmental toxins, and many other infectious diseases (Fan et al. 2016). Our lists of top outliers (supplementary tables 1–3, Supplementary Material online) do not closely match any particular previous scan for selection, though there is enrichment for repeat outliers (supplementary table 4, Supplementary Material online), including *LGALS8* (Andrés et al. 2009; DeGiorgio et al. 2014; Bitarello et al. 2018), *FBXO31* (Leffler et al. 2013), *SORD* (Tennessen and Akey 2011; DeGiorgio et al. 2014), and *DMBT1* (Leffler et al. 2013; DeGiorgio et al. 2014; Siewert and Voight 2017). One of the clearest signals of selection is on *PTK6* (fig. 3), a tyrosine-protein kinase involved in several cancer pathways. *PTK6* also shows parallel evolution at the variant level (Tennessen and Akey 2011) and is speculated to harbor adaptive polymorphisms impacting gastric bacterial infection (Jha et al. 2015). However, the specific selective pressure on *PTK6*, and most other outliers, is unknown.

Our outliers include numerous immune-related genes, which are especially prone to positive and balancing selection (Barreiro and Quintana-Murci 2010; Spurgin and Richardson 2010). This trend is consistent with selection via infectious agents from viruses to macroparasites. Many HLA-linked variants are outliers for *D_ng_* and *Π*_AHz_, but we have largely ignored these, as balancing selection on the HLA is already well documented (Spurgin and Richardson 2010). Excluding them, the *D_ng_* outliers are enriched for “positive regulation of immune response” (FDR = 6 × 10^−3^) and “antigen-binding” (FDR = 8 × 10^−5^). A majority of genes are shared between these two GO terms: the six immunoglobulin genes *IGHV3-23*, *IGHV1-3*, *IGLV2-14*, *IGKV2D-40*, *IGLV3-21*, and *IGHV1-24*, and dendritic cell receptor *CD209* (supplementary table 1, Supplementary Material online). Other immune relevant genes include interferon-inducible *IRGM*, T-cell surface glycoprotein *CD5*, and macrophage-expressed *CLEC4F*. Many of the immunity-related outliers encode transmembrane proteins, especially outliers for *D_ng_* and by a lesser extent *T*_R_. This pattern is consistent with selection for novelty in parasite-recognition proteins, leading to stable negative frequency-dependent selection (Divergent Haplotypes) or regular positive selection for new variants (Repeated Shifts). In contrast, for *Π*_AHz_ neither the exemplar loci (*HBB* and *G6PD*) nor most of the empirical outliers (supplementary table 3, Supplementary Material online) encode transmembrane proteins, and they show fewer direct ties to the immune system. Therefore, to the extent that Arrested Sweeps reflect selection by infectious agents, the adaptive response appears to compromise basic metabolic cytoplasmic proteins and thus prevent pathogens from rising to overwhelming levels, though perhaps at a cost to the host.

### Future Directions

The main goal of this study was to develop and evaluate statistical metrics for detecting malaria-associated signatures of selection. We used the 1000 Genomes as a reliable and comprehensive data set for this purpose, but future work on additional emerging data sets could further clarify patterns of selection (e.g., GenomeAsia100K Consortium 2019). These tests may be underpowered here due to variants being absent from the data set, including large indels and copy-number variants, but this issue can be addressed with more complete, high-coverage sets of genotypes. Furthermore, these methods are valid to apply to other species to detect signals of selection driven either by infection or by other factors. Scripts which calculate the statistics presented here are available at https://github.com/jacobtennessen/MalariaHallmarks (last accessed November 23, 2020). The identification and validation of additional examples of functional adaptive polymorphism will allow further refinement of tests for selection, leading to even more discoveries in a fortuitous feedback loop. In this way, the fields of evolutionary genetics and malaria pathology will continue to bolster each other as they have done for decades.

## Materials and Methods

### Divergent Haplotypes and *D_ng_*

For any pair of loci *i* and *j* with MAFs *p_i_* and *p_j_* and joint minor frequency *p_ij_*, one measure of LD between them (Kelly 1997) is defined as:

(1) δij=pij-pi*pj2pi*1-pi*pj*1-pj.

The mean LD for a set of *S* adjacent variants in *n* sequences (Kelly 1997) is thus:

(2) ZnS=2SS-1∑i=1S-1∑j=i+1Sδij.

For a target variant *j* and the *S* − 1 other variants *i* within distance *g* bp of *j*, in *n* sequences, the statistic *D_ng_* is defined here as:

(3) Dng=∑i=1S-1δij.

Because LD can be affected by population structure, *D_ng_* should be calculated for individual populations consistent with panmixia, and not across populations that differ in allele frequencies.

In order to evaluate *D_ng_*, we used the forward-time evolution simulation package SLiM (Messer 2013). We simulated genomic windows of 10,001 bp, with an overdominant balanced polymorphism in the center. *D_ng_* is not expected to only detect overdominance, which is only one type of balancing selection, but overdominance is logistically straightforward to simulate. By default, the dominance coefficient was 1e06 and the selection coefficient was 1e-08, yielding nearly identical homozygote finesses of effectively 1, a heterozygote fitness of 1.01 (=1 + 1e06 × 1e-08), and an expected MAF of 0.5. We also considered a “skew” scenario with uneven fitnesses: a dominance coefficient of 1.1 and a selection coefficient of 0.1, yielding homozygote fitnesses of 1 and 1.1, a heterozygote fitness of 1.11, and an expected MAF of 0.08. All other polymorphisms were selectively neutral and generated with mutation rate (μ) of 1e-07. We simulated either 50,000, 100,000, or 200,000 generation of evolution in a population of 10,000 individuals, with population-scaled recombination rate (ρ) set to either 0.01 or 0.001. We then calculated *D_ng_* for the balanced polymorphism, using *g* ranging from 100 to 5,000 bp. As a control, we simulated windows in which all polymorphisms were selectively neutral. These neutral control windows were 15,000 bp, and from each, we randomly chose a single variant for which to calculate *D_ng_*, with MAF ≥0.4 and at least 5,000 bp of sequence on either side. Other parameters matched the selection simulations, with one addition: we also considered a scenario with neutral evolution but a doubled μ of 2e-07, to test if elevated mutation rate alone can be distinguished from a signal of selection. For each distinct set of parameters, we ran 1,000 replicate simulations. We quantified overlap between the distributions of simulated windows by finding the lower *D_ng_* quantile in selection simulations that matched the equivalent upper quantile of neutral simulations.

We scanned for *D_ng_* in YRI in the 1000 Genomes data set (1000 Genomes Project Consortium 2015), using a distance of *g *=* *500 bp. We defined the top 50 candidate genes by ranking all protein-coding genes occurring outside of the HLA region (chromosome 6 between 29.7 and 33.1 Mb) based on *D_ng_* within exons or within 1 kb upstream of the gene, under the assumption that these sections are the most likely to harbor functional polymorphisms.

### Repeated Shifts and *T*_R_

Consider a set of three populations. For *R* nonoverlapping genomic windows of size *g* bp, excluding any windows with fewer than two variants, one calculates the highest *F*_ST_ among all variants, for each of the three pairwise comparisons. For each pairwise comparison, one then ranks all windows by *F*_ST_, using integers from 1 (highest *F*_ST_) to *R* (lowest *F*_ST_), such that higher *F*_ST_ values are ranked lower. Ties are rounded up; for example, if the highest *F*_ST_ value for a given population pair is observed in two different windows, both windows are assigned a rank of 2 and no window is assigned a rank of 1. Each window thus has three ranks, one for each population pair. For each window, the lowest rank *L* (most extreme *F*_ST_) and the highest rank *H* (least extreme *F*_ST_) are then identified. The statistic *T*_R_ is defined as:

(4) TR=L+H2R2.

As with Divergent Haplotypes, we performed forward-time simulations using SLiM. We simulated 10,000 unlinked genomic windows of 5,000 bp, with μ 1e-07, recombination rate 0 within windows, and all mutations selectively neutral. We allowed a population of 10,000 diploid individuals to evolve for 14,000 generations, at which point a second population of 5,000 individuals is generated from the first one. At 14,500 generations a third population of 5,000 individuals is generated from the second one, and all three populations continue to evolve for 500 more generations until the 15,000th generation. We then calculated *F*_ST_ values by randomly sampling 500 individuals per population. Furthermore, we simulated additional windows under the same parameters but with two adaptive mutations arising at generation 14,750: one in population 1 and one in population 3. For 1,000 of these windows, we used a selection coefficient of 0.005, and for 1,000 of these windows, we used a selection coefficient of 0.05. The adaptive mutations occur at different sites in the window and initially appear with 50 copies per population, to minimize the chance that they are lost to drift; this can be thought of as an existing rare neutral mutation suddenly becoming adaptive, or else a novel mutation occurring several generations earlier and reaching an abundance of 50 by generation 14,750. To calculate *T*_R_, we combined each adaptive window with the 10,000 neutral windows one at a time, rather than including all adaptive windows together, to simulate a genome in which the vast majority of windows are neutral. Thus, we could simultaneously evaluate whether the neutral windows behaved neutrally and whether the single adaptive window appeared as an outlier. We also considered a scenario with neutral evolution but a doubled μ of 2e-07, to test if elevated mutation rate alone can be distinguished from a signal of selection. Finally, to test for an effect of background selection, we simulated 10,000 windows with no positive selection but with a purifying selection coefficient of 0, −0.005, −0.05, or −0.5 occurring at 10% or 50% of sites (each combination of parameters represented equally), and also 1,000 windows each with positive selection (s = 0.05) on the target site and purifying selection (s = −0.05) at 10% or 50% of adjacent sites.

As with Divergent Haplotypes, we scanned the 1000 Genomes data set for *T*_R_ (1000 Genomes Project Consortium 2015). Our populations were Africa, Europe, and Asia. To maximize the signal of local adaptation and minimize admixture, we calculated African allele frequencies from all 504 individuals from the five sub-Saharan African populations (ESN, GWD, LWK, MSL, YRI) and ignored the two diaspora populations (ACB, ASW). For Europe, we used all 503 individuals from the five populations (CEU, TSI, FIN, GBR, IBS), and for East Asia, we used all 993 individuals from the ten South Asian and East Asian populations (CHB, JPT, CHS, CDX, KHV, GIH, PJL, BEB, STU, ITU). We used window sizes of *g *=* *5,000 bp and *g *=* *50,000 bp. We only examined autosomes to avoid the confounding effects of the X chromosome’s unique evolutionary rate impacting *F*_ST_. By default, we aligned windows beginning at the start of each chromosome. However, because windows are nonoverlapping, *T*_R_ is sensitive to how windows are aligned; two closely adjacent variants could be assigned to different windows and thus, their shared signal would be missed. Therefore, we also calculated *T*_R_ by starting windows at each 500-bp interval between 0 and 4,500 bp from the start of each chromosome. We defined the top 50 candidate genes by ranking protein-coding genes according to top *T*_R_ in windows overlapping exons.

### Arrested Sweeps and *Π*_AHz_

For a given variant with alleles *A* and *a*, one calculates the absolute difference in total heterozygous sites between individuals homozygous for allele *A* (*H*_AA_) and individuals homozygous for allele *a* (*H*_aa_). This difference can be quite large for rare variants, but these are uninteresting with respect to selection; instead, the signal of a sweep is a large difference for a variant that has risen beyond rarity (>1% frequency). Thus, the difference is multiplied by the MAF, which is calculated across the ingroup and designated *p_j_* as above. The product is the heterozygosity difference associated with allele *A*, *A*_H_, for which a high value indicates that one allele is associated with much more nucleotide diversity than the other, a signal of natural selection sweeping away variation in an otherwise polymorphic region:

(5) AH=Haa-HAA*pj.

The distribution of *F*_ST_ in the ingroup will depend on *p_j_* and on the particular populations examined and can extend below zero under Weir and Cockerham’s (1984) formula, but all that matters is the relative, not absolute, value of *F*_ST_. Thus, one ranks all ingroup *F*_ST_ values for variants with MAF of *p_j_* (rounded to the nearest 1% in practice), with higher *F*_ST_ values getting lower ranks as with *T*_R_. For each rounded *p_j_*:, ranks are divided by the total, yielding an *F*_ST_ rank proportion, *F*_R_, which ranges from 0 (high *F*_ST_) to 1 (low *F*_ST_, which is the relevant signal of selection in this scenario). The purifying selection metric is an adjusted reciprocal of MAF in the outgroup, *p*_o_, centered around an MAF of 1%. If *p*_o_ is 0, the adjusted reciprocal is 1 and does not change the final product. If *p*_o_ is 1%, the adjusted reciprocal is 0.5. As *p*_o_ increases above 1%, the adjusted reciprocal rapidly declines, indicating low evidence for an Arrested Sweep. Thus, the measure of a variant showing similar frequencies in the ingroup while excluded from the outgroup, *z*, is:

(6) z=FR*0.01po+0.01.

This product of these sweep and arrest metrics, *Π*_AHz_, is thus:

(7) ΠAHz=AH*z.

To calculate *Π*_AHz_ for a variant in a set of *n* phased diploid samples, one first calculates the total number of heterozygous sites for every possible diploid genome that could be formed from the 2*n* phased haploid genomes (2*n* choose two combinations). In practice, one can assume that the variant does not affect heterozygosity farther away than a given distance *g* (here set as 1 Mb) on the same chromosome, and thus heterozygosity can be calculated for a sufficiently large window on either side of the variant, rather than for the entire genome. This calculation can be performed once for a large genomic window (here set as 5 Mb) and then applied to all variants that are at least *g* from the edge of the window. For the target variant, one identifies all homozygotes for either allele among the 2*n*-choose-2 genomes and calculates the mean number of heterozygous sites for each, yielding *H*_aa_ and *H*_AA_. It is arbitrary which allele is designated as *A* versus *a*, and it does not depend on which is derived, dominant, etc. The absolute value of the difference between *H*_aa_ and *H*_AA_ is then multiplied by ingroup MAF *p_j_*, the *F*_ST_ rank *F*_R_, and the adjusted reciprocal of the outgroup MAF, (0.01/(*p*_o_ + 0.01). As with *D_ng_*, this test targets a single panmictic population in order to minimize the effect of population structure on LD. Additional populations are required to form the ingroup and outgroup.

As with the other statistics, we performed forward-time simulations using SLiM. We simulated genomic windows of 2,000,001 bp, with an asymmetrical overdominant balanced polymorphism in the center. As with *D_ng_*, overdominance is not the only form of balancing selection that *Π*_AHz_ could detect, but it is the representative form used in simulations. We considered “strong” and “weak” selection scenarios. In the “strong” scenario, selection against derived homozygotes was −0.9 and the dominance coefficient was −0.1, yielding genotype finesses of 1 (ancestral homozygote), 1.09 (heterozygote), and 0.1 (derived homozygote), and an expected MAF of 0.08. In the “weak” scenario, selection against derived homozygotes was −0.1 and the dominance coefficient was −0.1, yielding genotype finesses of 1 (ancestral homozygote), 1.01 (heterozygote), and 0.9 (derived homozygote), and an expected MAF of 0.08. All other polymorphisms were selectively neutral and generated with μ of 1e-07. We set ρ to 0.001. We first simulated 10,000 generations of neutral evolution in a population of 10,000 individuals, then the outgroup population of 10,000 individuals was generated from the initial population. After another 2,000 generations of neutral evolution, the adaptive mutation was generated in a single sample in the initial population. Unlike with *T*_R_, here it is important for the mutation to first appear in a single individual to generate the change in LD as the rare haplotype rapidly increases in frequency. After 100 additional generations, four new populations of 10,000 individuals were generated from the initial population to form the ingroup. The simulation then proceeded for 200 more generations to allow the ingroup populations to diverge. Thus, after a total of 12,300 generations, we calculated *Π*_AHz_ for the balanced polymorphism. As a control, we simulated windows in which all polymorphisms were selectively neutral. These neutral controls windows were 2,005,001 bp, and from each, we randomly chose a single variant for which to calculate *Π*_AHz_, with MAF ≥0.05 and at least 1 Mb of sequence on either side. Other parameters matched the selection simulations. We also considered neutral scenarios with reduced recombination (ρ = 0.0001) and increased mutation rate (μ = 2e-07) in order to test the effects of these nonadaptive processes. In some simulations, the target polymorphism was lost to drift while rare, but these were subsequently ignored. For each scenario, we examined 1,000 replicate simulations in which the target polymorphism was retained.

As with the other statistics, we scanned the 1000 Genomes data for *Π*_AHz_ (1000 Genomes Project Consortium 2015). We again used YRI as our target population. We used the five sub-Saharan African populations (ESN, GWD, LWK, MSL, YRI) as the ingroup under the assumption that all have experienced similar disease-induced selection. We used Europe (CEU, TSI, FIN, GBR, IBS) as the outgroup. We assumed that only variation within *g *=* *1 Mb was relevant to a given variant. We avoided all sequence within 5 Mb of the centromere on all chromosomes, because unusual levels of polymorphism and LD in these regions could swamp the signal. We only considered variants with *p_j_* of at least 0.01. We defined the top 50 candidate genes using the same criteria as for *D_ng_*.

### Synthesis

We searched for significant gene ontology (GO) terms with false discovery rate (FDR) values under 0.05 on http://geneontology.org (last accessed November 23, 2020). For all other tests for enrichment, we used Fisher’s exact tests. We tested for enrichment in the erythrocyte proteome using the genes detected by Bryk and Wiśniewski (2017). We also compared our top outliers against genes significantly associated with “erythroid cell” or “erythrocyte” in Medline abstracts based on *Z* score in a text mining database of genes and tissues (Santos et al. 2015; fig. 5 and supplementary tables 1–3, Supplementary Material online). Genome-wide, there are 3,613 genes with *Z* score ≥2 (852 “erythroid cell,” 3,495 “erythrocyte”) and 1,481 genes with *Z* score ≥3 (304 “erythroid cell,” 1,438 “erythrocyte”). As a control, we tested for enrichment in four other cell types: hepatocytes, neurons, epithelial cells, and endothelial cells. To look for malaria-associated polymorphisms, we compared our top outliers against the top loci for malaria susceptibility in three independent GWAS, regardless of whether they were significant. The first study (Timmann et al. 2012) reports 50 variants with *P* < 5e-05; we considered all genes within 100 kb of these variants, excluding genes adjacent to *HBB* and *ABO* as these adjacent genes are unlikely to be causal. The second study (Malaria Genomic Epidemiology Network 2019) reports 97 genomic regions overlapping variants with a Bayes factor >1,000; we considered all genes within these regions, excluding genes in the regions overlapping *HBB*, *ABO*, *GYPA/B/E*, and HLA, other than the exemplar loci themselves, as the other genes in those regions are unlikely to be causal. The third study (Milet et al. 2019) reports 28 genes with *P* < 1e-05; we considered all of these genes. To look for transmembrane domains, we used TMHMM v. 2.0 (Sonnhammer et al. 1998). To look for *Plasmodium*- or Piroplasm-interacting proteins, we used the curated list of Ebel et al. (2017). To look for low-recombination genes, we used a genetic map (Hinch et al. 2011) to calculate recombination rate in overlapping windows of 100–200 kb, and we defined low-recombination regions as those averaging less than 0.01 cM/Mb, which overlap 1,054 genes.

## Supplementary Material

Supplementary data are available at *Molecular Biology and Evolution* online.

## Supplementary Material

msaa294_Supplementary_Data
