# Supplementary material for: Three Signatures of Adaptive Polymorphism Exemplified by Malaria-Associated Genes
Source: Mol Biol Evol. 2020 Nov 13;38(4):1356–71. doi: 10.1093/molbev/msaa294 (PMC8042748; doi:10.1093/molbev/msaa294)
Supplement: msaa294_Supplementary_Data [file msaa294_supplementary_data.pdf]

Supplementary Material for:

Three signatures of adaptive polymorphism exemplified by malaria-associated genes

Jacob A. Tennessen and Manoj T. Duraisingh

Contents:

Supp Tables 1-4

Supp Figures 1-3

Supp Table 1. Top 50 genes based on D_{ng} in exons or within 1kb upstream, excluding the HLA region, along with the two exemplar loci. g = 500 bp.

Gene	Chromosome	D_{ng}	Transmembrane[a]	Erythrocyte-relevant[b]	Malaria GWAS[c]
ZNF99	19	34.97	no	no	no
SNX29	16	34.92	no	no	no
CYP2B6	19	32.56	no	E: 3.47	no
TMEM14C	6	28.77	yes	E: 2.12; EC: 3.84; Prot	no
KRTAP9-8	17	28.47	no	no	no
TNFRSF10D	8	26.81	yes	no	no
MUC4	3	25.34	yes	E: 2.01	no
TRAJ36	14	25.34	yes	no	no
SIRPA	20	23.13	yes	E: 4.84; EC: 2.37	no
CYP4F12	19	22.83	yes	no	no
NCMAP	1	21.72	yes	no	no
ZNF85	19	21.70	no	E: 2.37	no
MGAM	7	21.68	yes	E: 3.11	no
CD209	19	21.62	yes	E: 3.75	no
PPFIBP1	12	21.50	no	no	no
IGHV3-23	14[e]	21.34	yes	no	no
LGALS8	1	21.04	no	E: 2.75; Prot	no
PKD1L1	7	20.65	yes	no	no
SULT1A1	16	20.51	no	E: 2.33	no
IRGM	5	19.92	no	E: 2.56	no
IGLV3-21	22	19.29	yes	no	no
CFAP74	1	19.13	no	no	no
KIR3DL2	19	19.07	yes	E: 2.42	no
FAM86C1	11	18.98	no	no	no
AHNAK2	14	18.84	no	no	no
IGLV5-48	22	18.42	yes	no	no
IGLV2-14	22	18.18	yes	no	no
DHRS2	14	17.79	no	E: 2.54	no
MYO15B	17	17.69	no	no	no

ORC5	7	17.31	no	no	no
IGHV1-3	14[e]	16.69	yes	no	no
CHODL	21	16.55	yes	no	no
IGKV2D-40	2	16.51	yes	no	no
ZNF320	19	16.39	no	no	no
FCER2	19	16.34	yes	E: 2.74	no
MYOM1	18	16.31	no	no	no
PGPEP1L	15	16.29	no	no	no
CYB5R3	22	16.17	no	E: 4.34; EC: 2.48; Prot	no
CLEC4F	2	16.13	yes	E: 2.01	no
ZNF720	16	16.13	no	no	no
LRPPRC	2	16.09	no	no	no
DMBT1	10	16.08	no	no	no
MS4A12	11	15.98	yes	no	no
OR4L1	14[e]	15.96	yes	no	BF: 1090
OR51B6	11	15.87	yes	no	no
L1TD1	1	15.87	no	no	no
ARPC1B	7	15.77	no	Prot	no
IGHD2-21	14[e]	15.68	yes	no	no
IGHV1-24	14[e]	15.66	yes	no	no
CCDC158	4	15.51	no	no	no
ABO[d]	9	10.01[f]	yes	E: 2.77; EC: 2.42	BF: 1.2e+18; P: 4.3e-21
GYPA/B/E[d]	4	7.42[f]	yes	E: 6.93; EC: 6.64; Prot	BF: 3.9e+07

[a]via TMHMM (Sonnhammer et al. 1998)

[b]via text mining (Santos et al. 2015): E = Z-score (if over 2) for "erythrocyte" & EC = Z-score (if over 2) for "erythroid cell"; or via proteomics (Bryk and Wiśniewski 2017): Prot = in erythrocyte proteome

[c]evidence in genome-wide association study (GWAS): BF = Bayes factor via Malaria Genomic Epidemiology Network (2019) & P = p-value via Timmann et al. (2012)

[d]exemplar locus

[e]low-recombination region (Hinch et al. 2011)

[f]not in top 50

Supp Table 2. Top 50 genes based on T_R in exons or within 1kb upstream, along with the two exemplar loci. *g* = 5000 bp.

Gene	Chromosome	T_R	Transmembrane[a]	Erythrocyte-relevant[b]	Malaria GWAS[c]
PTK6	20	1.54e-08	no	no	no
SRMS	20	3.42e-07	no	no	no
FBXO31	16	8.85e-07	no	no	no
SPNS2	17	9.10e-07	yes	E: 3.64	BF: 3020
TTLL10	1[e]	1.03e-06	no	no	no
IGHG1	14[e]	7.02e-05	yes	Prot	no
SEMA4C	2	8.87e-06	yes	no	no
FAM178B	2	8.87e-06	no	no	no
MAP1LC3B	16	1.10e-05	no	E: 3.09	no
ZCCHC14	16	1.10e-05	no	no	no
OCA2	15	1.53e-05	yes	E: 2.94	no
CCDC92B	17	1.78e-05	no	no	no
PPDPF	20	1.87e-05	no	no	no
PRKAG3	2	2.95e-05	no	no	no
GSG1L2	17	3.11e-05	yes	no	no
SORD	15	3.35e-05	no	E: 3.82; Prot	no
SLC35F3	1	3.50e-05	yes	no	no
GLP2R	17	4.54e-05	yes	no	no
TMEM88B	1[e]	4.94e-05	yes	no	no
ANKRD65	1	4.94e-05	no	no	no
RSRP1	1	5.83e-05	no	no	no
RHD	1	5.83e-05	yes	E: 5.80; EC: 3.32; Prot	no
BRF1	14	7.23e-05	no	no	no
MLLT3	9	7.31e-05	no	E: 3.34; EC: 2.72	no
EEFSEC	3	7.76e-05	no	E: 2.91; Prot	no
CHRM5	15	9.22e-05	yes	no	BF: 1980
SNX20	16	9.47e-05	no	no	no
GALNT18	11	1.02e-04	yes	no	no
CNTN2	1	1.21e-04	no	no	no
SPIN1	9	1.25e-04	no	no	no

CNNM4	2	1.35e-04	yes	no	no
HELZ2	20	1.41e-04	no	no	no
FNDC11	20	1.41e-04	no	no	no
METTL7B	12	1.41e-04	yes	no	BF: 1030
ITGA7	12	1.41e-04	yes	no	BF: 1030
HERC2	15	1.50e-04	no	no	no
ANKRD39	2	1.55e-04	no	no	no
ANKRD23	2	1.55e-04	no	no	no
ARHGAP26	5	1.60e-04	no	no	no
CD5	11	1.60e-04	yes	E: 5.33; EC: 3.41	no
MYH9	22	1.69e-04	no	E: 3.61; Prot	no
MOGS	2	1.73e-04	yes	E: 2.13; Prot	no
MRPL53	2	1.73e-04	no	no	no
CCDC142	2	1.73e-04	no	no	no
PTPRM	18	1.73e-04	yes	no	P: 3.8e-08
SFTA3	14	1.74e-04	no	no	no
ADCYAP1R1	7	1.93e-04	yes	no	no
MYLK4	6	1.94e-04	no	no	P: 3.6e-07
TBC1D32	6	1.99e-04	no	no	no
MRPL20	1[e]	2.00e-04	no	no	no
ACKR1[d]	1	4.35e-04[f]	yes	E: 5.58; EC: 3.58; Prot	not in the included studies
CR1[d]	1	5.65e-03[f]	yes	E: 5.63; Prot	not in the included studies

[a]via TMHMM (Sonnhammer et al. 1998)

[b]via text mining (Santos et al. 2015): E = Z-score (if over 2) for "erythrocyte" & EC = Z-score (if over 2) for "erythroid cell"; or via proteomics (Bryk and Wiśniewski 2017): Prot = in erythrocyte proteome

[c]evidence in genome-wide association study (GWAS): BF = Bayes factor via Malaria Genomic Epidemiology Network (2019) & P = p-value via Milet et al. (2019)

[d]exemplar locus

[e]low-recombination region (Hinch et al. 2011)

[f]not in top 50

Supp Table 3. Top 50 genes based on Π_{AHz} in exons or within 1kb upstream, excluding the HLA region, along with the two exemplar loci.

Gene	Chromosome	Π_{AHz}	Transmembrane[a]	Erythrocyte-relevant[b]	Malaria GWAS[c]
RLIM	X	495.12	no	no	no
ABCB7	X[e]	272.91	yes	E: 3.64; EC: 4.75	no
HBB[d]	11	194.83	no	E: 6.36; EC: 5.42; Prot	BF: 1.8e+69; P: 5.6e-14
UGT2B10	4	187.49	yes	no	no
RABGAP1L	1[e]	162.56	no	Prot	no
ZDHHC15	X[e]	162.45	yes	no	no
ZKSCAN4	6	162.19	no	no	no
ZSCAN16	6[e]	153.98	no	no	no
ZKSCAN8	6	153.98	no	no	no
GABBR1	6	152.81	yes	E: 2.08	no
OR5V1	6	143.49	yes	no	no
TARDBP	1	141.49	no	Prot	no
RABGEF1	7[e]	141.35	no	Prot	no
CSMD3	8	139.00	yes	no	no
TESK2	1	136.32	no	no	no
EIF2B3	1	135.39	no	Prot	no
PRDX1	1	135.37	no	E: 3.72; Prot	no
UROD	1	130.54	no	E: 4.40; EC: 3.77; Prot	no
HECTD3	1	130.54	no	Prot	no
MAS1L	6	129.21	yes	no	no
OR2B3	6[e]	129.07	yes	no	no
ZSWIM5	1	128.42	no	no	no
ZSCAN12	6[e]	125.75	no	no	no
KRTAP4-4	17	125.66	no	no	no
DNAH14	1[e]	123.89	no	Prot	no
OIT3	10	122.99	no	no	no
NKAPL	6[e]	122.91	no	no	no
UGT2B4	4	122.69	yes	no	no
SLC9C2	1	122.20	yes	no	no

ABCC12	16	121.65	yes	no	no
SPAG16	2	121.48	no	no	no
ZSCAN23	6	121.30	no	no	no
ZBTB37	1	119.20	no	no	no
SLC38A4	12	118.48	yes	no	no
ANKRD45	1	116.10	no	no	no
KRT32	17	114.66	no	Prot	no
MOG	6	113.55	no	E: 3.94	no
CNTLN	9	113.05	no	no	no
QRICH1	3	112.89	no	no	no
VKORC1L1	7	111.13	yes	Prot	no
PLA2G12B	10	106.82	no	no	no
KRTAP2-2	17	106.65	no	no	no
NIPBL	5[e]	105.43	no	EC: 2.82	BF: 3170
CPLANE1	5	105.33	no	no	no
MBNL3	X	103.46	no	no	no
GPX2	14	103.42	no	E: 3.90	no
RC3H1	1[e]	102.85	no	no	no
KRT35	17	101.02	no	Prot	no
KDM3A	2	100.31	no	Prot	no
KRTAP2-3	17	99.97	no	no	no
G6PD[d]	X	68.80[f]	no	E: 5.68; Prot	not in the included studies

[a]via TMHMM (Sonnhammer et al. 1998)
[b]via text mining (Santos et al. 2015): E = Z-score (if over 2) for "erythrocyte" & EC = Z-score (if over 2) for "erythroid cell"; or via proteomics (Bryk and Wiśniewski 2017): Prot = in erythrocyte proteome
[c]evidence in genome-wide association study (GWAS): BF = Bayes factor via Malaria Genomic Epidemiology Network (2019) & P = p-value via Timmann et al. (2012)
[d]exemplar locus
[e]low-recombination region (Hinch et al. 2011)
[f]not in top 50

Supp Table 4. Overlap between outliers in this study and those from other genome-wide scans for selection.

Study	Genes[a]	D_{ng} genes	T_R genes	Π_{AHz} genes	Enrichment[b]
Voight et al. 2006	271	none	*MYH9*	*CSMD3, DNAH14*	1.5
Andrés et al. 2009	28	*LGALS8*	none	none	4.9
Tennessen and Akey 2011	1388	*ARPC1B, CYB5R3, LRPPRC, MUC4, ZNF99*	*ARHGAP26, CNTN2, EEFSEC, GLP2R, HERC2, MLLT3, MYH9, OCA2, PTK6, PTPRM, SLC35F3, SORD, ZCCHC14*	*ABCC12, CSMD3, RC3H1, SLC38A4, ZSWIM5*	2.4***
Leffler et al. 2013	442	*DMBT1 ,PKD1L1*	*FBXO31, OCA2*	*SPAG16*	1.6
DeGiorgio et al. 2014	149	*DMBT1, LGALS8, LRPPRC, PKD1L1, ZNF85*	*MYLK4, SORD*	none	6.3***
Ferrer-Admetlla et al. 2014	33	*PKD1L1*	none	none	4.1
Siewert and Voight 2017	1381	*DHRS2, DMBT1, MGAM, MYO15B*	*GLP2R, PTPRM, SLC35F3*	*CSMD3, MOG, SPAG16, UGT2B4*	1.1
Bitarello et al. 2018	502	*CCDC158, CYP4F12, IRGM, LGALS8, NCMAP, OR4L1*	*CHRM5, CNTN2, SLC35F3*	none	2.5*
Cheng and DeGiorgio 2019	25	none	none	*SPAG16*	6.0

[a]Number of reported outlier genes or genes in reported outlier regions. For scans conducted on separate populations, only results from Africans or African Americans are included.
[b]Enrichment for shared genes between our results and each study. *p < 0.05; ***p<0.001

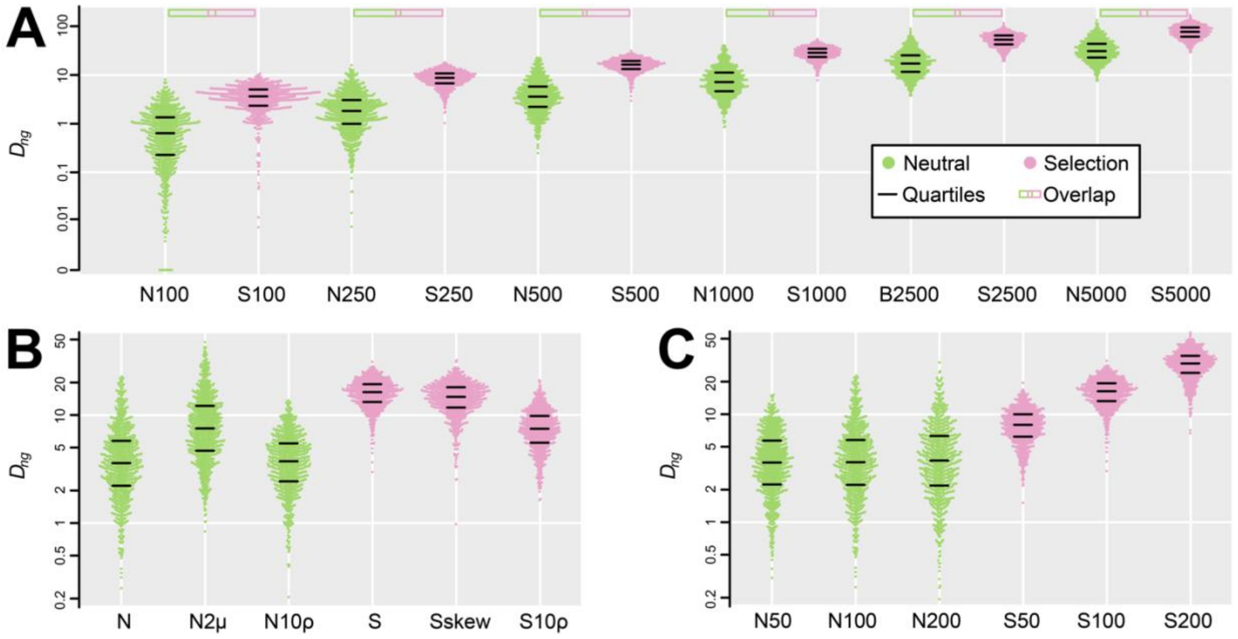

Supplementary Figure 1. Simulation results for D_{ng}. (A). Distributions of D_{ng} values for neutral and selection scenarios with varying g values, with ρ = 0.001, expected MAF = 0.5, μ = 1e-7, and 100,000 generations. Distributions are labeled N ("neutral") or S ("selection"), along with g value (e.g. N250 = neutral simulations with g = 250 bp). Boxes above each pair of neutral and selection distributions indicate the degree of overlap between the distributions. Overlap is minimized for intermediate g values of 500 and 1000 bp (lower 7% S quantile meets upper 7% N quantile). (B) Distributions of D_{ng} values for neutral and selection scenarios with g = 500 bp and 100,000 generations. "N" and "S" are the same as "N500" and "S500" in (A), and the remaining distributions have the same parameters except for a single alteration each. "N2μ" has a doubled mutation rate, "N10ρ"and "S10ρ" have 10-fold increased recombination rate, and "Sskew" has expected MAF of 0.08. (C). Distributions of D_{ng} values with g = 500bp, ρ = 0.001, expected MAF = 0.5, μ = 1e-7, and age of the balanced polymorphism either 50,000 (N50 and S50), 100,000 (N100 and S100) or 200,000 (N200 and S200) generations.

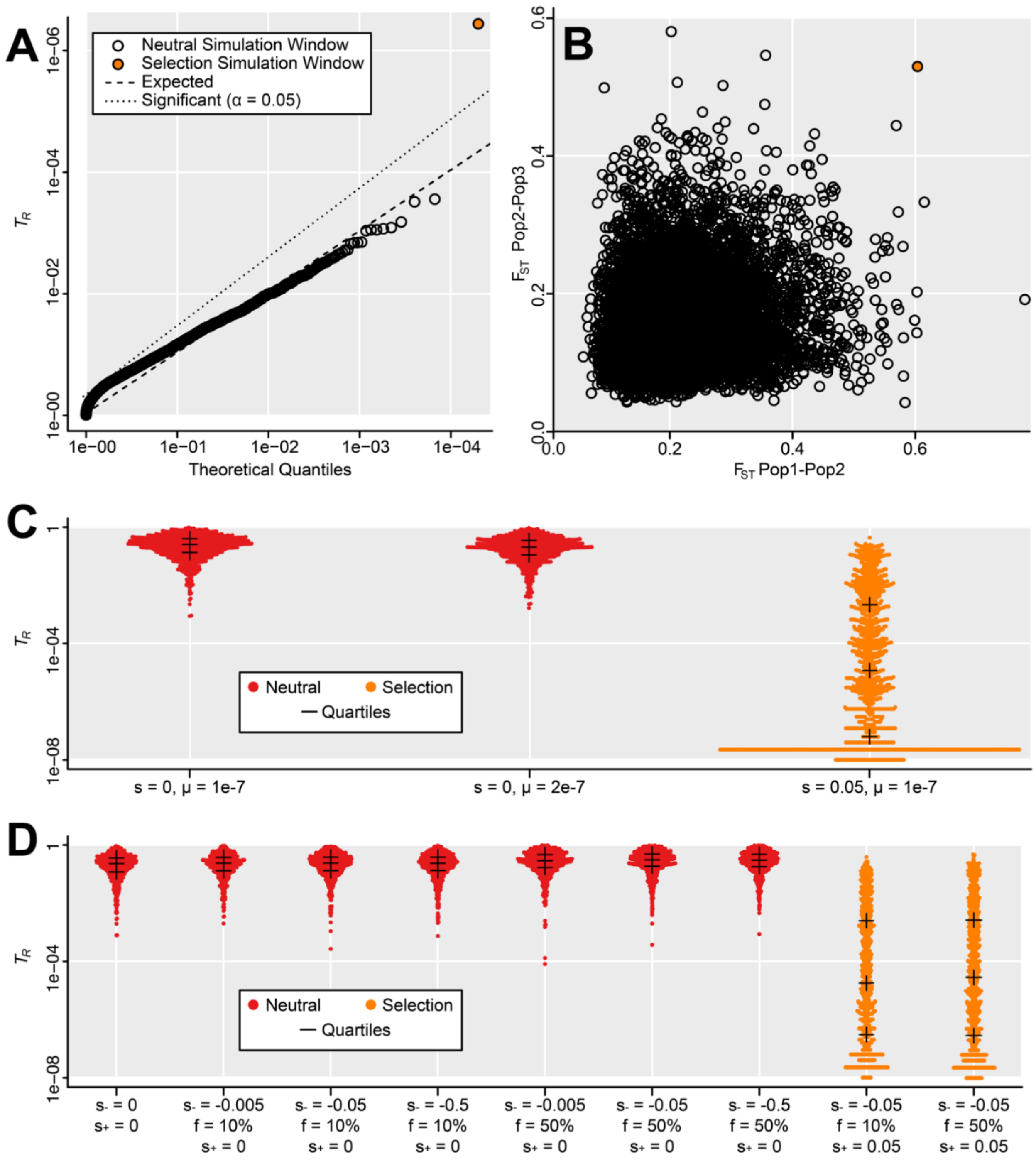

Supplementary Figure 2. Simulation results for T_R with strong selection (s = 0.05). (A) Out of 10,000 neutral windows and a single selection window, the selection window is the clear significant outlier in a QQ plot. (B) In this case, the selection outlier does not show the highest F_{ST} in any comparison, yet it is an extreme outlier for T_R which combines across F_{ST} values. (C) Distribution of T_R across 1000 windows each for neutral evolution (s = 0, μ = 1e-7), neutral evolution with elevated mutation rate (s = 0, μ = 2e-7), and strong selection (s = 0.05, μ = 1e-7), when each

window is included alone among 10,000 neutral windows. T_R is substantially lower when strong selection operates. Under weak selection (s = 0.005, not shown), the distribution of T_R (median = 0.25) is similar to neutrality (median = 0.26). (D). Background selection has little effect on T_R. In the absence of positive selection ("neutral", red), with four negative selection coefficients (s- = 0, -0.005, -0.05, or -0.5) occurring at either 10% or 50% (f), T_R is robust to the degree of background selection. T_R is very slightly elevated under background selection (median = 0.24 for f of 10%; 0.30 for f of 50%) relative to no selection (median = 0.23), indicating that background selection cannot explain the enrichment for low T_R in genes (Figure 3B). If a single adaptive window ("selection", orange) is included showing both positive selection (s+ = 0.05) and background selection (s- = -0.05, f = 10% or 50%), it typically shows much lower T_R than windows with only background selection, demonstrating that T_R has high power to detect adaptation even in the presence of background selection.

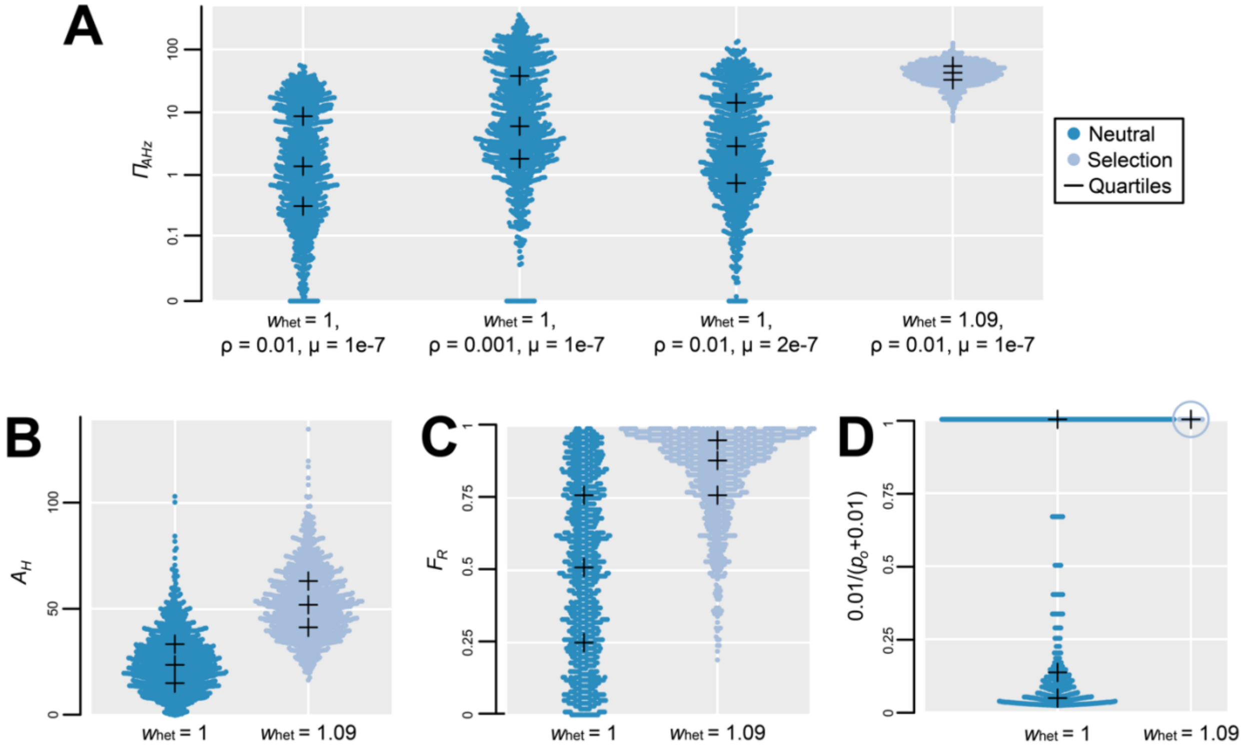

Supplementary Figure 3. Simulation results for Π_{AHz} with strong selection (w_{het} = 1.09). (A) Distributions of Π_{AHz} values for four evolutionary scenarios (left to right): neutral evolution, neutral evolution with reduced recombination, neutral evolution with increased mutation rate, and strong selection with heterozygote fitness w_{het} = 1.09. (B) Distributions of the difference in total heterozygous sites times ingroup allele frequency (A_H) for neutral evolution and strong selection, both with ρ = 0.01, μ = 1e-7. (C) Distributions of F_{ST} rank proportion (F_R) for neutral evolution and strong selection, both with ρ = 0.01, μ = 1e-7. (D) Distributions of the adjusted reciprocal of the outgroup allele frequency (0.01/(p_o+0.01) for neutral evolution and strong selection, both with ρ = 0.01, μ = 1e-7; selection scenario is always at one because p_o is zero, so datapoints are overlapped and circled for ease of visualization. Under weak selection (w_{het} = 1.01, not shown), the distribution of Π_{AHz} (median = 10) is higher than neutrality (median = 1) but the difference is modest.
